# Glycaemic control and insulin therapy are significant confounders of the obesity paradox in patients with heart failure and diabetes mellitus

**DOI:** 10.1007/s00392-023-02268-3

**Published:** 2023-08-22

**Authors:** Hanna Fröhlich, Anna Bossmeyer, Syed Kazmi, Kevin M. Goode, Stefan Agewall, Dan Atar, Morten Grundtvig, Norbert Frey, John G. F. Cleland, Lutz Frankenstein, Andrew L. Clark, Tobias Täger

**Affiliations:** 1 Department of Cardiology, Angiology, and Pulmology, University Hospital Heidelberg, Im Neuenheimer Feld 410, 69120 Heidelberg, Germany; 2 Hull University Teaching Hospitals NHS Trust, Hull, UK; 3 Department of Cardiology, and Institute of Clinical Medicine, Oslo University Hospital Ulleval, University of Oslo, Oslo, Norway; 4 Medical Department, Innlandet Hospital Trust Division Lillehammer, Lillehammer, Norway; 5 National Heart and Lung Institute, Royal Brompton and Harefield Hospitals, Imperial College, London, UK; 6 Robertson Centre for Biostatistics and Clinical Trials, Glasgow, UK

**Keywords:** Obesity paradox, Reverse epidemiology, Heart failure, Diabetes mellitus, Mortality

## Abstract

**Background:**

A high body mass index (BMI) confers a paradoxical survival benefit in patients with heart failure (HF) or diabetes mellitus (DM). There is, however, controversy whether an obesity paradox is also present in patients with HF and concomitant DM. In addition, the influence of glycaemic control and diabetes treatment on the presence or absence of the obesity paradox in patients with HF and DM is unknown.

**Methods:**

We identified 2936 patients with HF with reduced ejection fraction (HFrEF) in the HF registries of the universities of Heidelberg, Germany, and Hull, UK (general sample). Of these, 598 (20%) were treated for concomitant DM (DM subgroup). The relationship between BMI and all-cause mortality was analysed in both the general sample and the DM subgroup. Patients with concomitant DM were stratified according to HbA1c levels or type of diabetes treatment and analyses were repeated.

**Results:**

We found an inverse BMI-mortality relationship in both the general sample and the DM subgroup. However, the obesity paradox was less pronounced in patients with diabetes treated with insulin and it disappeared in those with poor glycaemic control as defined by HbA1c levels > 7.5%.

**Conclusion:**

In patients with HFrEF, a higher BMI is associated with better survival irrespective of concomitant DM. However, insulin treatment and poor glycaemic control make the relationship much weaker.

**Graphical abstract:**

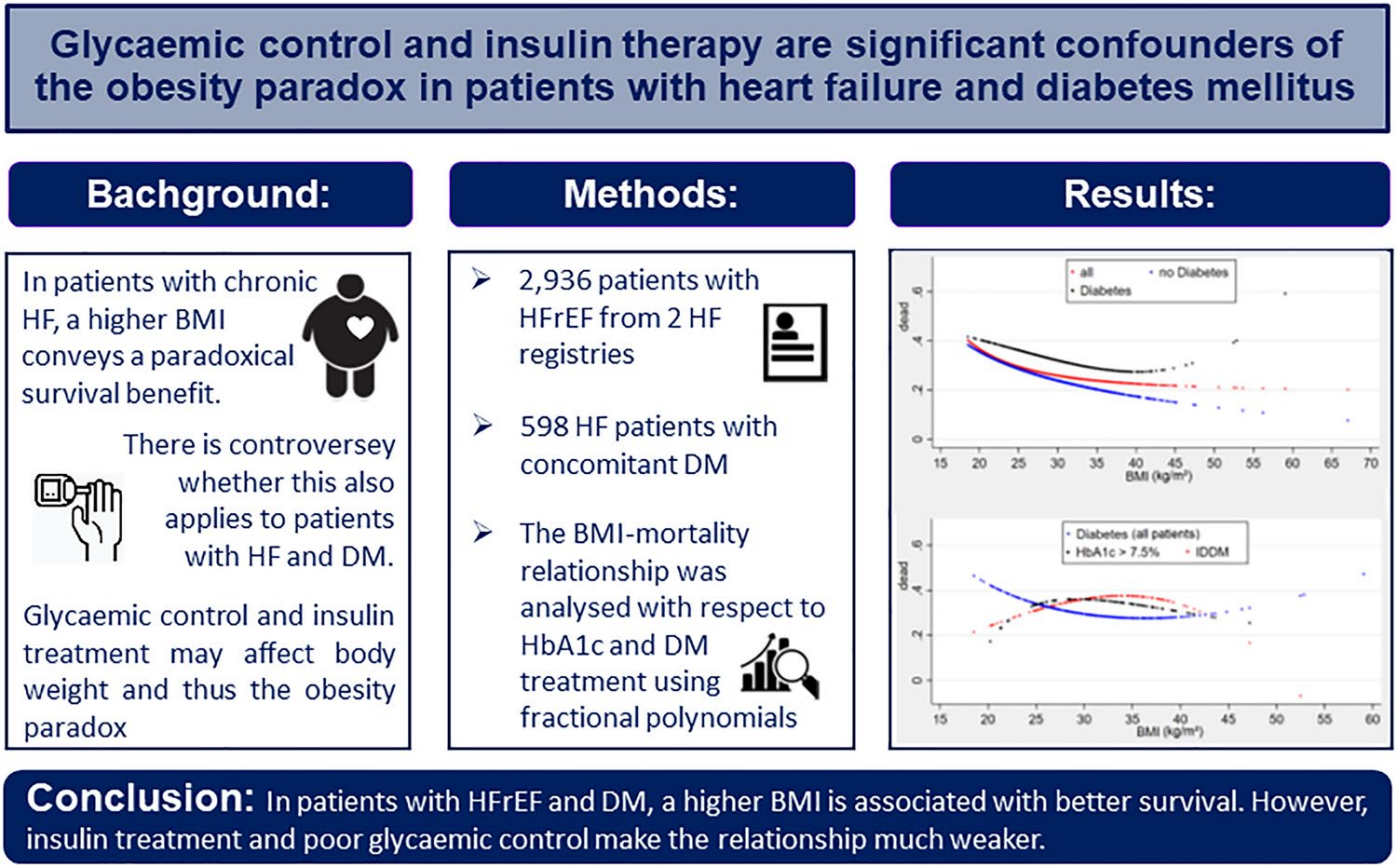

**Supplementary Information:**

The online version contains supplementary material available at 10.1007/s00392-023-02268-3.

## Introduction

Obesity is an established risk factor for the development of cardiovascular (CV) disease and heart failure (HF), and it adversely affects CV haemodynamics, structure, and function. However, a high body mass index (BMI) is associated with better outcomes in patients with established CV disease such as coronary heart disease, stroke or heart failure (HF) [1–5]. This counterintuitive association has been termed the “obesity paradox”. Besides CV disease, the obesity paradox has been observed in a wide range of clinical conditions including end-stage renal failure, advanced cancer, chronic obstructive pulmonary disease and diabetes mellitus (DM) [6–12]. The obesity paradox has been specifically demonstrated in patients with HF with consistent results seen across geographical locations, sex, age range, and the presence or absence of comorbidities such as hypertension or atrial fibrillation [13]. However, there is controversy whether an obesity paradox also exists in patients with chronic HF who suffer from concomitant DM. While some cohort studies reported absence of the obesity paradox in patients with HF and DM [5, 14–18], others found an inverse relationship between BMI and mortality irrespective of concomitant DM [19–23].

The cause of the conflicting observations is unknown. However, a significant interaction between BMI and glycaemic control is a possible reason, with obese patients being susceptible to insulin resistance and hyperglycaemia [24, 25]. In addition, in patients with DM, BMI may also be modified by diabetes treatment. While insulin therapy may increase body weight, oral antidiabetics such as metformin or sodium-glucose cotransporter 2 inhibitors may cause weight loss. The presence or absence of an obesity paradox in patients with HF and concomitant DM may thus depend on glycaemic control and type of diabetes treatment. We, therefore, explored the relationship between BMI and mortality in a multi-national cohort of ambulatory patients with chronic HF and DM with regard to glycaemic control and type of diabetes treatment.

## Methods

### Patient selection and follow-up

We retrospectively selected patients with chronic HF with reduced ejection fraction (HFrEF) from the HF registries of the universities of Heidelberg, Germany, and Hull, UK. Patients attending the community HF clinics of the Department of Academic Cardiology, University of Hull, UK, or the University Hospital Heidelberg, Germany, since December 1995 for evaluation of HF were offered inclusion into the local HF registries. Recruitment was prospective and continuous for each database and centre. Both databases reflect all-comer cohorts. For each registry, patients were included after stabilization of both clinical status and medication. Since both university hospitals are providers of secondary and tertiary care, the registries reflect a broad representation of patients of their respective regions. All patients gave their written informed consent for data storage and evaluation. The study conformed to the principles outlined in the Declaration of Helsinki and was approved by the local ethics committees.

The diagnosis of HFrEF was established according to guidelines on the basis of typical symptoms and signs associated with an objective abnormality of cardiac structure or function on echocardiography, cardiac magnetic resonance imaging, or left heart catheterisation [26]. All included patients had a left ventricular ejection fraction (LVEF) ≤ 45%. Baseline characteristics included medical history, physical examination, BMI, LVEF, blood chemistry, and medication. BMI was calculated according to the formula body weight (kg)/(height (m))^2^. Medication was at the discretion of the referring physician. For the purpose of the present study, only patients with available information on LVEF and BMI were selected (general sample).

In the general sample, a subgroup of patients with concomitant DM was identified. Only patients with DM treatment at the time of enrolment into the HF registries were included (DM subgroup).

Surviving patients were followed for a minimum of six months. Determination of survival status and follow-up was performed by scheduled visits to the outpatient clinic, by telephone calls either to the patients’ homes or to their physicians, or by electronic hospital records. For the purpose of the present analysis, patients were censored as “alive” at the date of this last contact. Five-year all-cause mortality was the predefined endpoint of the study.

### Statistical analysis

The data are presented as mean ± SD, median (interquartile range), or number (%) as appropriate. To compare frequencies, chi-squared analysis was performed. To test for significant differences between two groups, the two-sample Mann–Whitney test and Student’s *t* test were used where appropriate. All tests are two-tailed and an arbitrary *p* value of less than 5% was regarded as statistically significant.

In order to analyse the influence of BMI on mortality, fractional polynomials were modelled using the STATA statistical software. Analyses were repeated in the DM subgroup after stratification of patients according to their type of diabetes treatment (insulin dependent DM [IDDM] vs. non-insulin dependent DM [NIDDM]) or HbA1c levels: HbA1c < 6.5% (very good glycaemic control), HbA1c 6.5–7.5% (treatment goal for patients with DM), and HbA1c > 7.5% (poor glycaemic control) [27–30]. In a second step, fractional polynomials were used to analyse the relationship between HbA1c levels and five-year mortality. Analyses were repeated in the DM-subgroup after stratification of patients according to their BMI following the WHO classification: BMI 18.5–25 kg/m^2^ (normal weight) vs. 25–30 kg/m^2^ (overweight) vs. > 30 kg/m^2^ (obesity). Patients with a BMI < 18.5 kg/m^2^ (n = 31) were excluded from analyses since cachexia is associated with a poor prognosis irrespective of the underlying disease [31–33]. Finally, analyses were repeated after adjustment for age, sex, LVEF, NT-proBNP, renal function and HF treatment.

## Results

We identified 2936 patients with HFrEF and available information on BMI and comorbidities in both HF registries (general sample). Of these, 598 (20.4%) patients received treatment for concomitant DM at the time of inclusion into the HF registries (DM subgroup). Baseline characteristics of the general sample and of the DM subgroup are shown in Table 1. Patients in the DM subgroup differed from patients in the general sample in a number of variables. For example, HF patients with concomitant DM were older, had a higher BMI and were more likely to suffer from symptomatic HF of ischaemic origin as compared to HF patients without concomitant DM. In addition, the proportion of patients with guideline recommended HF therapy was higher in patients with concomitant DM. Baseline characteristics of HF patients with DM stratified by diabetes treatment (IDDM vs. NIDDM) are presented in eTable 1.

**Table 1:** Baseline characteristics of patients with HFrEF stratified by presence or absence of concomitant DM

	General sample* (*n* = 2936)	No DM (*n* = 2052)	DM subgroup** (*n* = 598)	*p* value
Age, years	62 ± 13	62 ± 14	66 ± 10	< *0.001*
Female, *n* (%)	643 (22)	457 (22)	117 (20)	0.28
Height, cm	172 ± 9	172 ± 9	172 ± 9	0.36
Weight, kg	84 ± 18	82 ± 17	90 ± 19	< *0.001*
BMI, kg/m^2^	28.3 ± 5.2	27.5 ± 4.9	30.5 ± 5.5	< *0.001*
SBP, mmHg	122 ± 22	121 ± 21	126 ± 24	< *0.001*
Heart rate, 1/min	71 ± 14	71 ± 14	73 ± 13	< *0.001*
Aetiology, *n* (%)				< *0.001*
Ischaemic	1683 (57)	1076 (52)	430 (72)	
Non-ischaemic	1253 (43)	976 (48)	168 (28)	
NYHA, *n* (%)				< *0.001*
I	802 (27)	617 (30)	130 (22)	
II	1204 (41)	864 (42)	232 (39)	
III	878 (30)	542 (26)	224 (38)	
IV	42 (1)	21 (1)	10 (2)	
LVEF, %	31 ± 9	32 ± 9	31 ± 9	0.09
6MWT, m	421 ± 144	433 ± 143	381 ± 140	< *0.001*
Creatinine, mg/dL (mmol/L)	1.1 (0.9–1.4) 97 (80–124)	1.1 (0.9–1.3) 97 (80–115)	1.2 (1.0–1.5) 106 (88–133)	< *0.001*
eGFR, ml/min*1.73m^2^	68 (50–89)	71 (53–91)	62 (45–82)	< *0.001*
Sodium, mmol/L	139 ± 3	139 ± 3	138 ± 3	< *0.001*
Potassium, mmol/L	4.4 ± 0.5	4.4 ± 0.5	4.5 ± 0.5	< *0.001*
NT-proBNP, ng/L	851 (278–2234)	790 (246–2123)	948 (318–2273)	< *0.001*
Glucose, mg/dL (mmol/L)	110 ± 46 (6.11 ± 2.55)	96 ± 19 (5.33 ± 1.05)	149 ± 72 (8.27 ± 4.0)	< *0.001*
HbA1c, %	6.2 ± 1.0	5.7 ± 0.4	7.1 ± 1.3	< *0.001*
Cholesterol, mg/dL	180 ± 44	186 ± 43	164 ± 43	< *0.001*
Comorbidity, *n* (%)				
Hypertension	1718 (59)	1126 (55)	401 (67)	< *0.001*
COPD	324 (11)	217 (10)	77 (13)	0.22
Atrial fibrillation	268 (9)	184 (9)	56 (9)	0.90
Smoker, *n* (%)				< *0.001*
Ever	1890 (64)	1349 (66)	340 (57)	
Never	1040 (35)	699 (34)	257 (43)	
Medication				
ACEI/ARB/ARNI, *n* (%)	2511 (86)	1720 (84)	541 (90)	< *0.001*
Beta blocker, *n* (%)	2341 (80)	1606 (78)	506 (85)	< *0.001*
MRA, *n* (%)	1341 (46)	863 (42)	307 (51)	< *0.001*
Loop diuretic, *n* (%)	1836 (63)	1178 (57)	447 (75)	< *0.001*
Digitalis, *n* (%)	733 (26)	479 (24)	159 (28)	0.21
Insulin, *n* (%)	242 (8)	0 (0)	242 (40)	< *0.001*
Antidiabetic drugs, *n* (%)***	424 (14)	0 (0)	424 (71)	< *0.001*
Aspirin, *n* (%)	1097 (37)	720 (35)	283 (47)	*0.001*
Anticoagulation, *n* (%)	1247 (42)	860 (42)	250 (42)	0.91

Significant *p* values are written in italics

*HFrEF* heart failure with reduced ejection fraction, *DM* diabetes mellitus, *n* number, *BMI* body mass index, *SBP* systolic blood pressure, *HR* heart rate, *NYHA* New York Heart Association functional class, *LVEF* left ventricular ejection fraction, *6MWT* 6 min walk test, *eGFR* estimated glomerular filtration rate using the Chronic Kidney Disease Epidemiology Collaboration formula [46], *HbA1c* haemoglobin A1c, *COPD* chronic obstructive pulmonary disease, *ACEI* angiotensin converting enzyme inhibitor, *ARB* angiotensin receptor blocker, *ARNI* angiotensin receptor neprilysin inhibitor, *MRA* mineralocorticoid receptor antagonist

*Only patients with recorded diabetes state, BMI and a LVEF < 45% at the time of inclusion into the HF registries were selected. **Only patients with treatment for DM at the time of inclusion into the HF registries were selected. Patients with a diagnosis of (pre)diabetes not receiving any treatment for DM were excluded. ***Details of anti-diabetic treatment in the subset of patients included in the Heidelberg HF registry are presented in the supplemental material

Median follow-up was 5.9 (3.2–9.3) years in the general sample and 5.6 (3.1–8.5) years in the DM subgroup, respectively. During that time, 1255 (52%) and 284 (47%) patients died.

**Fig. 1:**
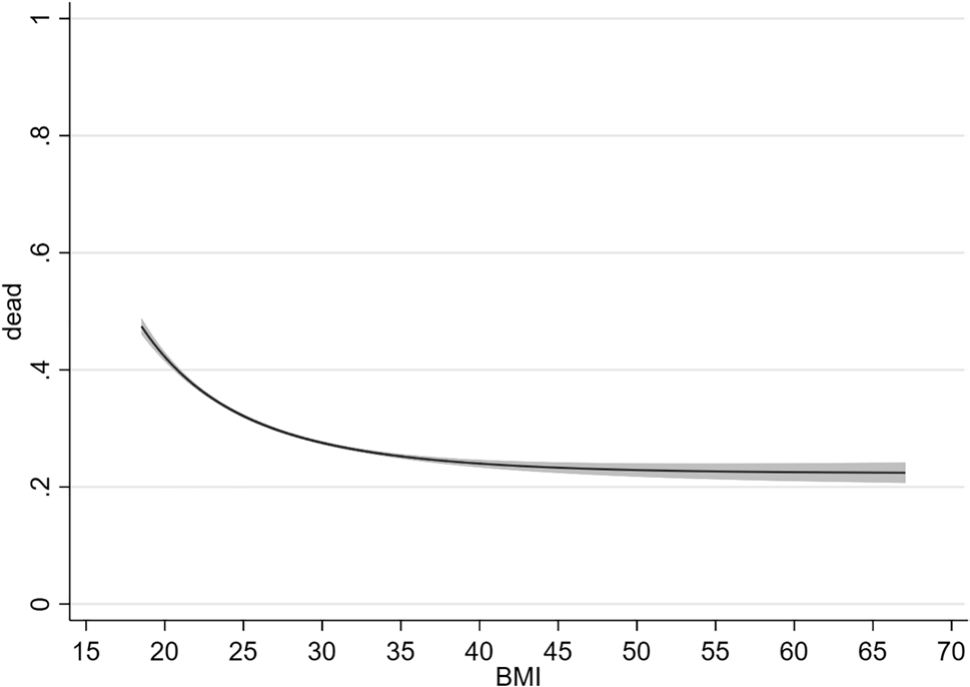
Relationship between BMI and 5-year all-cause mortality in ambulatory patients with chronic stable HFrEF (general sample). *BMI* body mass index (kg/m^2^), *HFrEF* heart failure with reduced ejection fraction

**Fig. 2:**
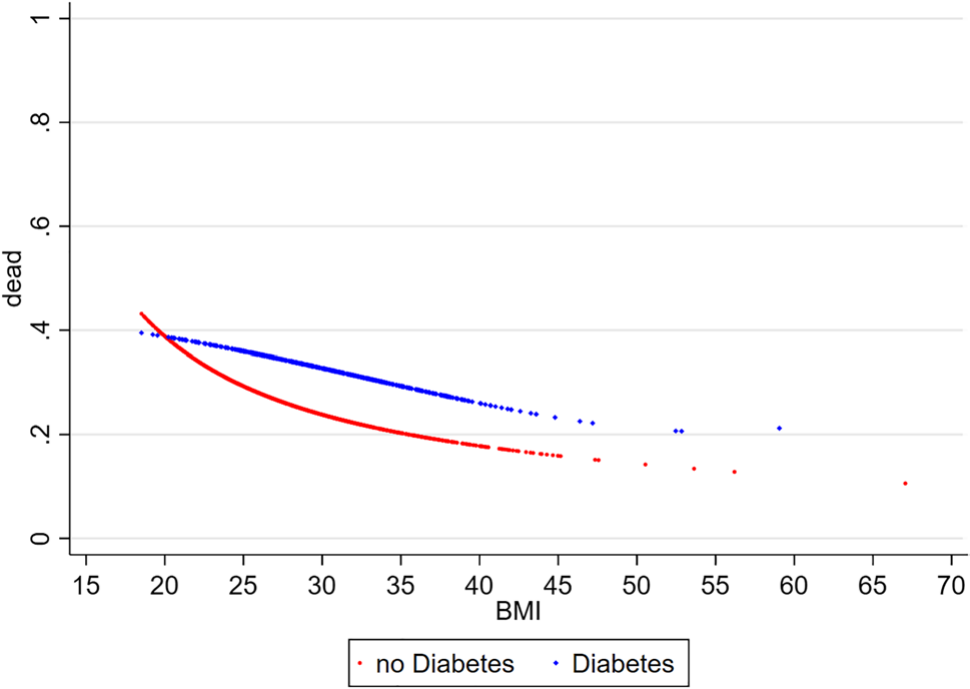
Relationship between BMI and 5-year all-cause mortality in ambulatory patients with chronic stable HFrEF with or without concomitant DM. *BMI* body mass index (kg/m^2^), *DM* type 2 diabetes mellitus, *HFrEF* heart failure with reduced ejection fraction

In the general sample, there was an inverse relationship between BMI and all-cause mortality. As shown in Fig. 1, the 5-year risk of death was doubled in lean HF patients as compared to obese subjects. The obesity paradox was similarly present in patients with HFrEF with or without concomitant DM (Fig. 2). However, mortality was generally higher in the DM subgroup as compared to HF patients without concomitant DM.

**Fig. 3:**
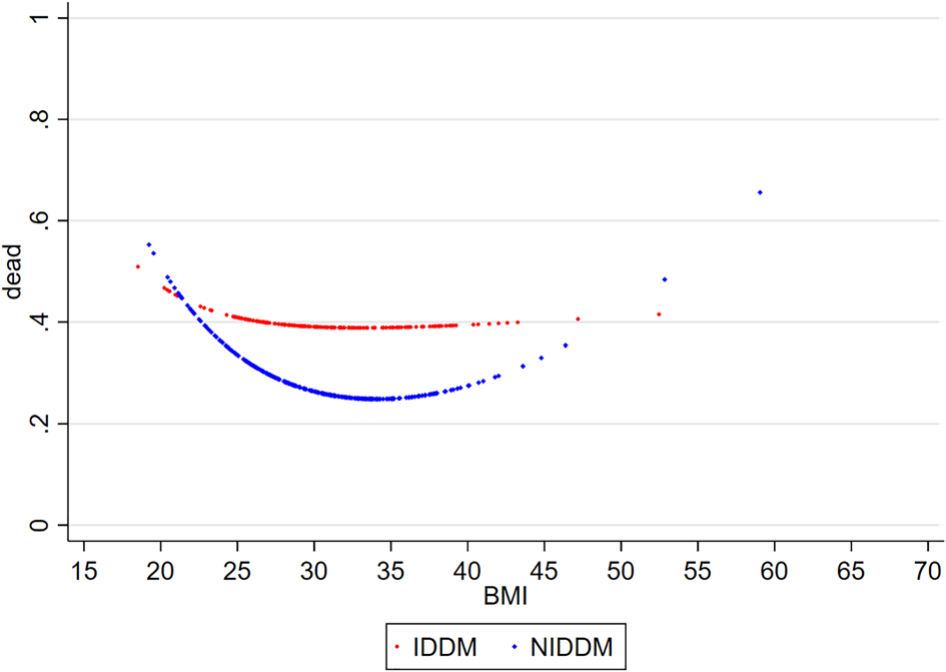
Relationship between BMI and 5-year all-cause mortality in ambulatory patients with chronic stable HFrEF and concomitant DM (diabetes subgroup) stratified by type of diabetes treatment. *BMI* body mass index (kg/m^2^), *DM* type 2 diabetes mellitus, *HFrEF* heart failure with reduced ejection fraction, *IDDM* insulin dependent diabetes mellitus, *NIDDM* non insulin dependent diabetes mellitus

When patients were stratified by the type of diabetes treatment, the obesity paradox was clearly present in patients with NIDDM: there was a U-shaped relationship between BMI and 5-year mortality, with obesity stage I (BMI 30–35 kg/m^2^) being associated with the lowest mortality (Fig. 3). Our analysis also suggests that there was a U-shaped relationship between BMI and 5-year mortality in patients with IDDM. However, the obesity paradox was substantially weaker in patients receiving insulin treatment. In addition, IDDM was associated with higher mortality than NIDDM in overweight and obese patients but not in lean subjects.

**Fig. 4:**
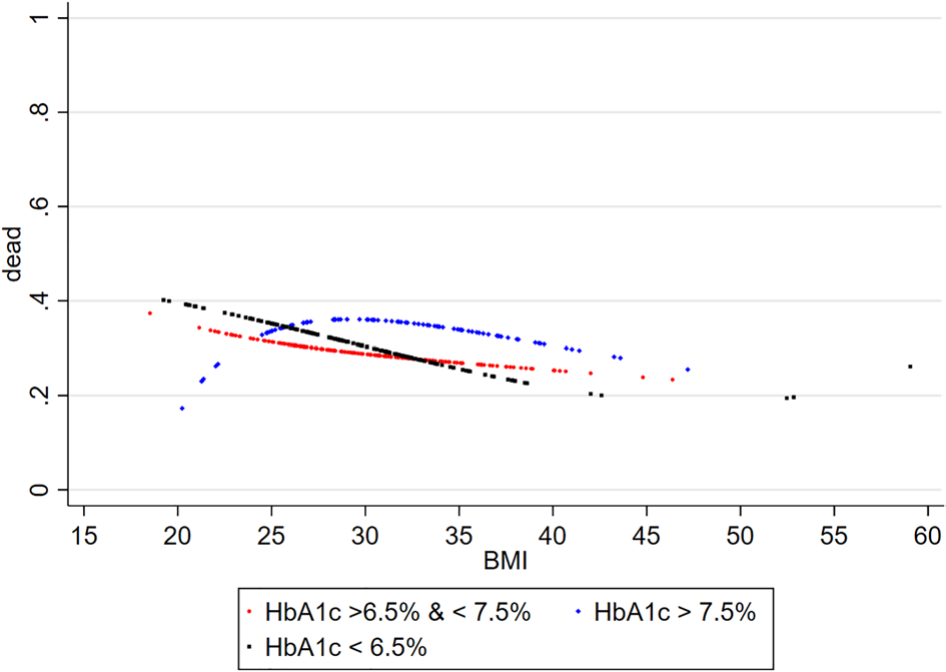
Relationship between BMI and 5-year all-cause mortality in ambulatory patients with chronic stable HFrEF and concomitant DM (diabetes subgroup) stratified by HbA1c. *BMI* body mass index (kg/m^2^), *DM* type 2 diabetes mellitus, *HFrEF* heart failure with reduced ejection fraction

When patients were stratified by HbA1c levels, there was an inverse relationship between BMI and 5-year all-cause mortality in patients with excellent or adequate glycaemic control (HbA1c < 6.5% and 6.5–7.5%, respectively) but not in those with poor glycaemic control (HbA1c > 7.5%) (Fig. 4). In the latter group, mortality in overweight patients was higher than in lean or obese subjects. While HbA1c levels > 7.5% were associated with higher mortality as compared to HbA1c levels < 7.5% in patients with BMI > 25 kg/m^2^, this was not true in lean patients.

**Fig. 5:**
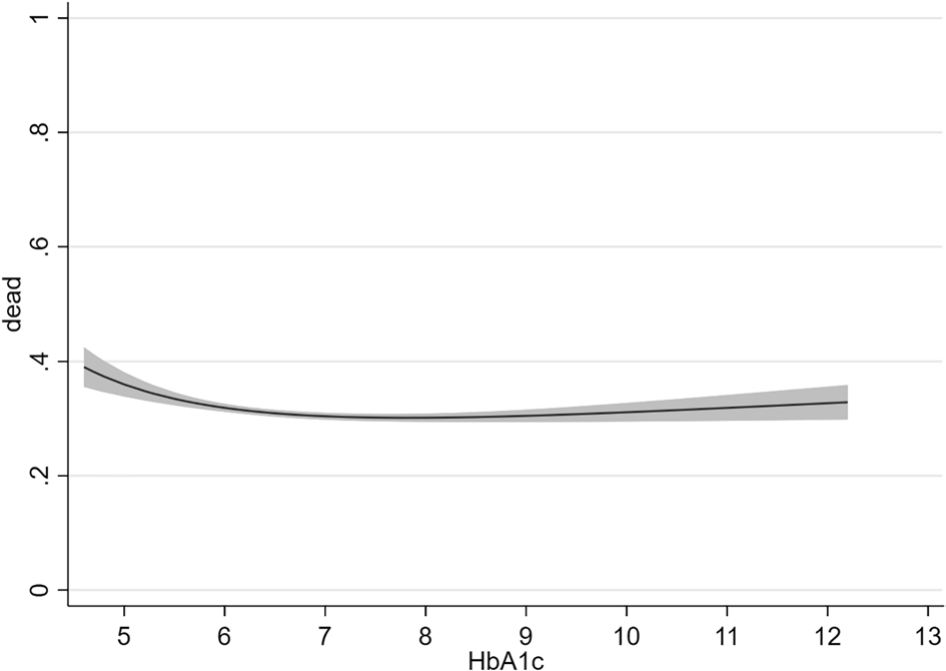
Relationship between HbA1c (%) and 5-year all-cause mortality in ambulatory patients with chronic stable HFrEF and concomitant DM (diabetes subgroup). *DM* type 2 diabetes mellitus, *HFrEF* heart failure with reduced ejection fraction

**Fig. 6:**
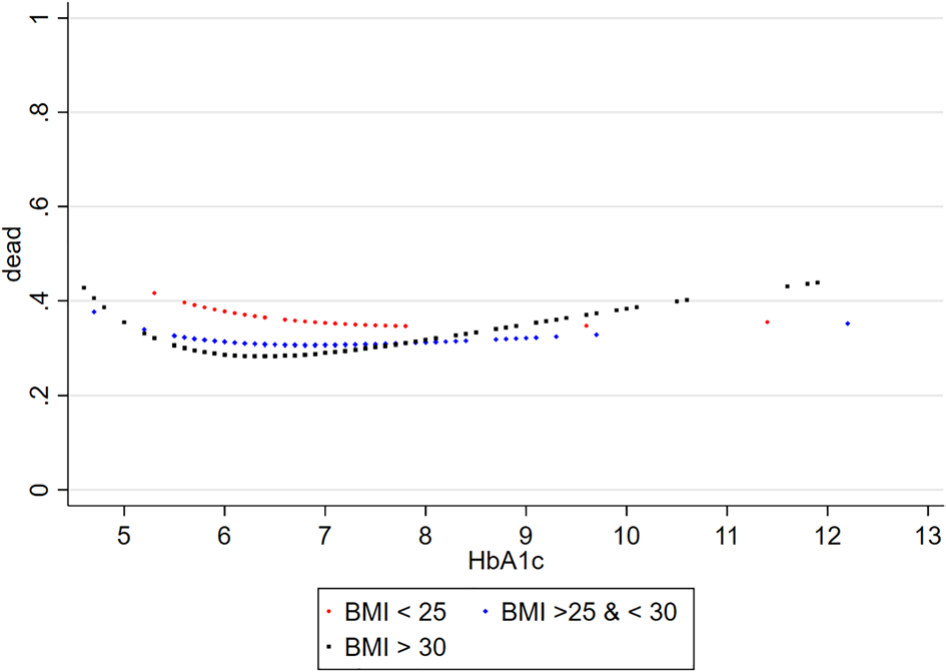
Relationship between HbA1c (%) and 5-year all-cause mortality in ambulatory patients with chronic stable HFrEF and concomitant DM (diabetes subgroup) stratified by BMI. *BMI* body mass index (kg/m^2^), *DM* type 2 diabetes mellitus, *HFrEF* heart failure with reduced ejection fraction

In the DM subgroup, we found a U-shaped relationship between HbA1c levels and 5-year mortality (Fig. 5). Mortality was lowest in patients with HbA1c concentrations ranging from ≈ 6.5 to 7.5% and slightly increased in patients with very low (< 5.5%) or very high (> 10%) HbA1c levels. When stratified by BMI categories, the U-shaped relationship was most pronounced in obese patients (Fig. 6).

Analyses adjusted for age, sex, LVEF, NT-proBNP, renal function and HF treatment are presented in eFigs. 1–6. After adjustment for confounders, an obesity paradox was still present irrespective of the presence or absence of concomitant DM (eFigs. 1, 2). However, the relationship between BMI and mortality changed to a U-shaped pattern and it was attenuated in patients with HF and DM. Stratification by type of diabetes treatment and HbA1c confirmed the significant confounding effect of insulin therapy and glycaemic control on the obesity paradox (eFigs. 3, 4). In addition, multivariable analyses confirmed the U-shaped relationship between HbA1c levels and 5-year mortality (eFigs. 5, 6). However, multivariable adjustment resulted in large confidence intervals and should, therefore, be interpreted with caution.

## Discussion

We have found an inverse relationship between BMI and all-cause mortality in a multinational cohort of ambulatory patients with chronic HFrEF with or without concomitant DM. In patients with HF and DM, however, glycaemic control and type of diabetes treatment emerged as significant confounders of the obesity paradox, with no benefit from high BMI in patients with insulin treatment or poor glycaemic control.

Since the first description of a paradoxical survival benefit with high BMI for patients with chronic heart failure, a number of studies and meta-analyses have confirmed the association in acute and chronic HF [3, 34, 35]. However, whether there is an obesity paradox in patients with HF and concomitant DM in uncertain. Zamora et al. reported no obesity paradox in 1102 ambulatory patients with HF and DM [17]. Similarly, there was no advantage of obesity in a small cohort of 52 patients with HF and metabolic syndrome [15]. In contrast, the ARIC study showed a protective effect of overweight and obesity among 1487 patients with incident HF irrespective of concomitant DM [21]. Obese patients with DM included in the Worcester Heart Failure Study had 20–40% lower odds of dying within 5 years after hospitalisation for HF than normal weight patients [22].

One reason for the conflicting findings in prior studies may be the wide array of datasets included in analyses. In particular, patient populations differed significantly with respect to definitions of HF and DM. Prior studies may also be sensitive to assumptions regarding the estimation sample and functional form for BMI [36]. Because the relationship between BMI and mortality is nonlinear and the distribution of BMI is right skewed, the vast majority of prior studies have employed a non-parametric approach by treating BMI as a categorical variable [14, 16, 17, 19, 20, 22]. This approach, however, loses information and power because it assumes that all individuals in a certain category exhibit the same mortality risk [36]. Categorisation is particularly problematic if groups are large because it increases the risk of heterogeneity within a category. In addition, the position and number of cut-off points is arbitrary.

A number of studies, therefore, have used other approaches such as Cox regression analyses that maintain BMI as a continuous variable [14, 16–18, 20]. However, Cox regression analyses may be inadequate if the proportional hazard assumption is violated. Our study is the first to address the relationship between BMI and mortality in patients with established chronic HFrEF and DM using fractional polynomials. The fractional polynomials method maintains BMI as a continuous variable and it allows the data to determine the best fitting functional form for BMI. It is thus a flexible approach to modelling the nonlinear and asymmetric relationship between mortality and BMI and it may improve the model fit compared to other commonly employed models [36].

In the present study, an inverse BMI–mortality relationship was similar in HF patients with or without concomitant DM. However, after multivariable adjustment, the obesity paradox was attenuated in the diabetes subgroup. This finding partly explains the previous conflicting reports on the presence or absence of an obesity paradox in patients with HF and DM since previous studies controlled for confounders in various ways. In the present study, mortality was generally higher in the DM subgroup which reflects the well-known negative impact of DM on prognosis. The underlying pathophysiological mechanisms of the obesity paradox remain controversial. Potential reasons for an increased mortality in lean patients include cachexia, frailty, reduced muscle mass and muscle strength, reduced metabolic reserve, and a greater burden of chronic disease and comorbidities [3, 8, 11, 37–40]. With regard to DM, multiple studies have shown an association between lean diabetes, malnutrition in the early years of life and poor socioeconomic status. The key pathophysiological feature appears to be a defect in insulin secretory capacity as opposed to peripheral insulin resistance as noted in classical type 2 DM [41].

Stratification by type of diabetes treatment showed a U-shaped association between BMI and mortality in patients with NIDDM, whereas insulin treatment significantly altered the BMI–mortality relationship in both univariable and multivariable analyses. While mortality was higher in lean patients with DM irrespective of type of treatment, the survival benefit of being overweight or obese was substantially reduced. This finding points towards a significant interaction between insulin treatment and body weight. Studies have reported that a 3 to 9 kg insulin-associated weight gain occurs in the first year of initiating insulin therapy, predominantly caused by adipose tissue. In the present study, BMI was significantly higher in patients with IDDM as compared to NIDDM. Insulin-associated weight gain has been associated with a worse cardio-metabolic risk profile, with higher total and LDL cholesterol levels, renal glomerular hyperfiltration and increased systolic blood pressure [42]. Because BMI is correlated with both body fat and lean mass, the survival advantage of higher BMI may result from higher lean mass rather than increased adipose tissue. This potential misclassification of obesity in terms of body composition may partially explain the observed association between weight status and mortality in patients with HF.

In the present study, poor glycaemic control as defined by HbA1c levels > 7.5% was associated with greater survival in lean patients when compared to patients with BMI > 25 kg/m^2^ or those with HbA1c levels < 7.5%. We have no conclusive explanation for this counterintuitive observation. The number of HF patients with BMI < 25 kg/m^2^ and HbA1c levels > 7.5%, however, was limited and results should, therefore, be interpreted with caution. The majority of patients with poor glycaemic control were overweight or obese. In these patients, a trend towards an inverse BMI–mortality relationship similar to that observed in other HbA1c categories was noted. Further research is needed to clarify the BMI-mortality relationship in patients with HFrEF and poorly controlled DM.

Current guidelines advice a HbA1c target < 7.0% to prevent vascular complications in patients with DM [43]. In younger patients with a short duration of DM and no evidence of cardiovascular disease, more stringent goals may be considered, whereas less-stringent HbA1c targets may be adequate for elderly patients with long-standing DM and multiple comorbidities. Our data argue for a moderate HbA1c target (6.5–7.5%) in patients with HFrEF and DM. This target may especially be appropriate for patients with obesity since the U-shaped relationship between HbA1c and mortality was most pronounced in this subgroup. When compared to BMI, however, the absolute impact of glycaemic control on mortality was relatively small.

## Limitations

The intrinsic limitations of any post-hoc analysis should be considered in this study. Patients’ BMI was calculated from weights and heights abstracted from their medical records at the time of inclusion into the HF registries. We did not have information on subsequent changes in patients’ weight during follow-up. This issue may be particularly important because individual BMI trajectories are often characterized by high variability. However, inclusion into the present study was performed after stabilisation of both patients’ clinical status and medication in an ambulatory setting. Weight measurements used for the present analyses therefore likely represent patients’ dry weight. In addition, we did not have data available on alternative anthropometric indices of obesity such as waist circumference. In previous studies, however, high BMI and high waist circumference were similarly associated with better outcomes in a cohort of patients with advanced systolic HF, lending further support for an obesity paradox in HF [44]. The diagnosis of DM, treatment regimens and HbA1c measurements were captured at baseline only. Fluctuations of HbA1c levels and changes in diabetes treatment during follow-up were not considered. In addition, details on anti-diabetic treatment were available in a subset of patients only. Therefore, further subgroup analyses with respect to specific oral anti-diabetic treatments were not possible. Some patients in the non-diabetes group may have developed DM during follow-up which may have resulted in misclassification of patients. Lastly, our analyses are restricted to the endpoint all-cause mortality. Therefore, we cannot comment on the presence or absence of the obesity paradox with respect to other outcomes of interest such as cardiovascular mortality. However, a meta-analysis of six studies involving 22,807 patients with chronic HF confirmed the obesity paradox with regard to cardiovascular mortality and hospitalisations [45].

## Conclusion

The present study confirms the presence of an obesity paradox in a multinational cohort of 2936 patients with chronic stable HFrEF with or without concomitant DM. However, the obesity paradox was much less marked in patients with DM treated with insulin and it disappeared in those with poor glycaemic control as defined by HbA1c levels > 7.5%. Additional research is warranted to advise patients with HFrEF and DM in their weight management. Future studies should particularly aim to clarify HbA1c and weight targets in HF patients with insulin treatment or poor glycaemic control.

### Supplementary Information

Below is the link to the electronic supplementary material.

Supplementary file1 (DOCX 368 KB)

## Data Availability

The data that support the findings of this study are available from the corresponding author, [LF], upon reasonable request.

## Code availability

N.A.
