# Supplementary material for: Glycaemic control and insulin therapy are significant confounders of the obesity paradox in patients with heart failure and diabetes mellitus
Source: Clin Res Cardiol. 2023 Aug 22;113(6):822–30. doi: 10.1007/s00392-023-02268-3 (PMC11108876; doi:10.1007/s00392-023-02268-3)
Supplement: Supplementary file 1 — Supplementary file1 (DOCX 368 KB) [file 392_2023_2268_MOESM1_ESM.docx]

**eTable 1:** Baseline characteristics of patients with HFrEF and concomitant DM stratified by type of diabetes treatment

|  | **DM subgroup** (n=598)** | **IDDM (n=242)** | **NIDDM (n=356)** | **p-value** |
| --- | --- | --- | --- | --- |
| **Age, *years*** | 66 ± 10 | 64 ± 10 | 66 ± 10 | *0.02* |
| **Female, *n (%)*** | 117 (20) | 57 (24) | 60 (17) | *0.04* |
| **Height, *cm*** | 172 ± 9 | 171 ± 9 | 172 ± 9 | 0.76 |
| **Weight, *kg*** | 90 ± 19 | 91 ± 18 | 89 ± 19 | 0.18 |
| **BMI, *kg/m²*** | 30.5 ± 5.5 | 30.9 ± 5.4 | 30.1 ± 5.5 | 0.08 |
| **SBP, *mmHg*** | 126 ± 24 | 123 ± 22 | 127 ± 25 | *0.02* |
| **Heart rate, *1/min*** | 73 ± 13 | 72 ± 12 | 73 ± 14 | 0.72 |
| **Aetiology, *n (%)*** |  |  |  | 0.71 |
| **ischaemic** | 430 (72) | 176 (73) | 254 (71) |  |
| **non-ischaemic** | 168 (28) | 66 (27) | 102 (29) |  |
| **NYHA, *n (%)*** |  |  |  | 0.62 |
| **I** | 130 (22) | 50 (21) | 80 (22) |  |
| **II** | 232 (39) | 89 (37) | 143 (40) |  |
| **III** | 224 (38) | 97 (40) | 127 (36) |  |
| **IV** | 10 (2) | 5 (2) | 5 (1) |  |
| **LVEF, *%*** | 31 ± 9 | 31 ± 9 | 32 ± 9 | 0.28 |
| **6MWT, *m*** | 381 ± 140 | 375 ± 140 | 384 ± 141 | 0.55 |
| **Creatinine, *mg/dL (µmol/L)*** | 1.2 (1.0-1.5)  (107 (88-133)) | 1.2 (1.0-1.6)  (107 (88-141)) | - 1. (0.9-1.4)   (97 (80-124)) | *0.004* |
| **eGFR, *ml/min*1.73m²*** | 62 (45-82) | 58 (43-77) | 65 (48-84) | *0.004* |
| **Sodium, *mmol/L*** | 138 ± 3 | 138 ± 3 | 138 ± 3 | 0.98 |
| **Potassium, *mmol/L*** | 4.5 ± 0.5 | 4.4 ± 0.5 | 4.5 ± 0.5 | 0.32 |
| **NT-proBNP, *ng/L*** | 948  (318-2,273) | 1,003  (273-2,637) | 920  (339-2,115) | 0.67 |
| **Glucose, *mg/dL*** | 149 ± 72 | 166 ± 87 | 138 ± 57 | *<0.001* |
| **HbA1c, *%*** | 7.1 ± 1.3 | 7.6 ± 1.5 | 6.7 ± 0.9 | *<0.001* |
| **Cholesterol, *mg/dL*** | 164 ± 43 | 162 ± 46 | 165 ± 41 | 0.67 |
| **Comorbidity, *n (%)*** |  |  |  |  |
| **Hypertension** | 401 (67) | 173 (71) | 228 (64) | 0.06 |
| **COPD** | 78 (13) | 30 (12) | 47 (13) | 0.59 |
| **Atrial fibrillation** | 56 (9) | 16 (7) | 40 (11) | *0.04* |
| **Smoker, *n (%)*** |  |  |  | *<0.001* |
| **ever** | 340 (57) | 120 (51) | 219 (62) |  |
| **never** | 257 (43) | 121 (49) | 137 (38) |  |
| **Medication** |  |  |  |  |
| **ACEI/ ARB/ ARNI, *n (%)*** | 541 (94) | 217 (95) | 325 (94) | 0.80 |
| **Beta blocker, *n (%)*** | 506 (85) | 219 (90) | 287 (81) | *<0.001* |
| **MRA, *n (%)*** | 307 (51) | 130 (54) | 177 (50) | 0.36 |
| **Loop diuretic, *n (%)*** | 447 (75) | 184 (76) | 263 (74) | 0.57 |
| **Digitalis, *n (%)*** | 159 (28) | 69 (30) | 90 (26) | 0.30 |
| **Insulin, *n (%)*** | 242 (40) | 242 (100) | 0 (0) | *<0.001* |
| **Antidiabetic drugs, *n (%)*** | 424 (71) | 68 (28) | 356 (100)* | *<0.001* |
| **Aspirin, *n (%)*** | 283 (47) | 120 (50) | 163 (46) | 0.40 |
| **Anticoagulation, *n (%)*** | 250 (42) | 101 (42) | 149 (42) | 1.00 |
| Significant p-values are written in italics.  *In 182 patients with NIDDM included in the Heidelberg HF registry, oral anti-diabetic treatment was: acarbose: 10 (5%), DPP4 inhibitor: 16 (9%), repaglinide: 6 (3%), metformin: 126 (69%), and sulfonylurea: 52 (29%).  HFrEF, heart failure with reduced ejection fraction; DM, diabetes mellitus; IDDM, insulin dependent diabetes mellitus; NIDDM, non-insulin dependent diabetes mellitus; n, number; BMI, body mass index; SBP, systolic blood pressure; HR, heart rate; NYHA, New York Heart Association functional class; LVEF, left ventricular ejection fraction; 6MWT, 6 minute walk test; eGFR, estimated glomerular filtration rate using the Chronic Kidney Disease Epidemiology Collaboration formula (38); HbA1c, haemoglobin A1c; COPD, chronic obstructive pulmonary disease; ACEI, angiotensin converting enzyme inhibitor; ARB, angiotensin receptor blocker; ARNI, angiotensin receptor neprilysin inhibitor; MRA, mineralocorticoid receptor antagonist. | | | | |

**eFig. 1** Relationship between BMI and 5-year all-cause mortality in ambulatory patients with chronic stable HFrEF after multivariable adjustment


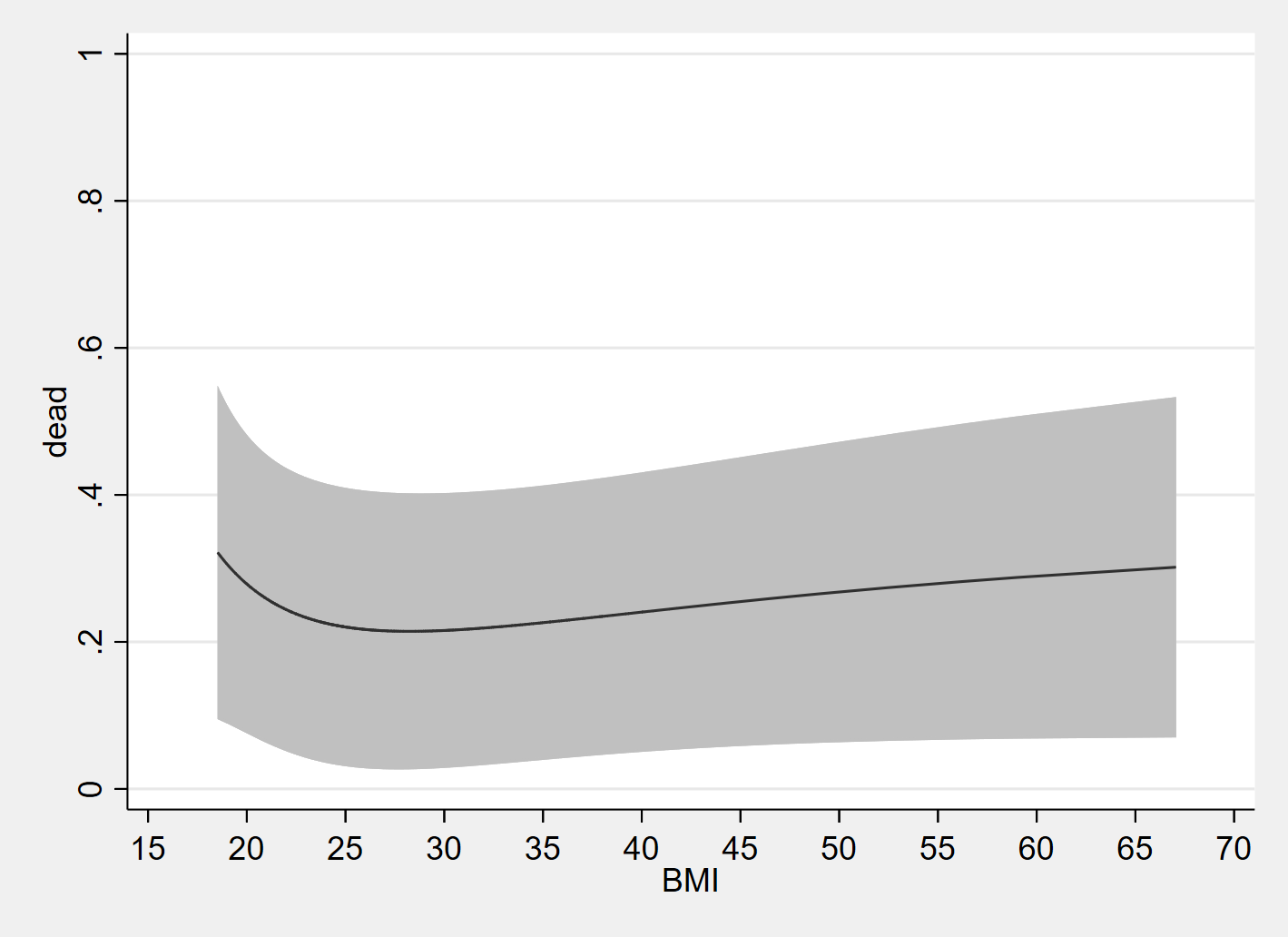


Legend: BMI, body mass index (kg/m²); HFrEF, heart failure with reduced ejection fraction

**eFig. 2** Relationship between BMI and 5-year all-cause mortality in ambulatory patients with chronic stable HFrEF with or without concomitant DM after multivariable adjustment


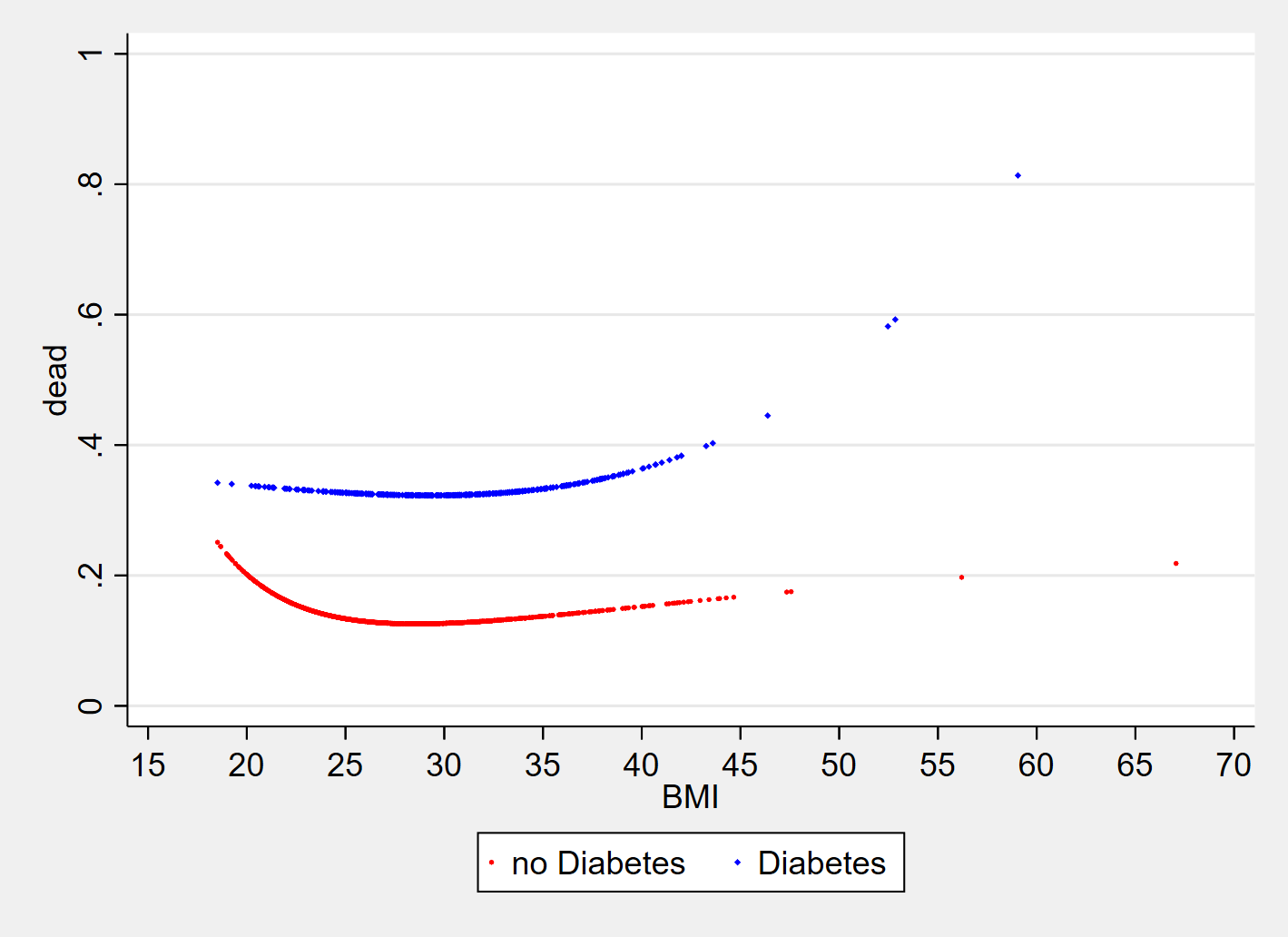


Legend: BMI, body mass index; DM, type 2 diabetes mellitus; HFrEF, heart failure with reduced ejection fraction.

**eFig. 3** Relationship between BMI and 5-year all-cause mortality in ambulatory patients with chronic stable HFrEF and concomitant DM (diabetes subgroup) stratified by type of diabetes treatment after multivariable adjustment


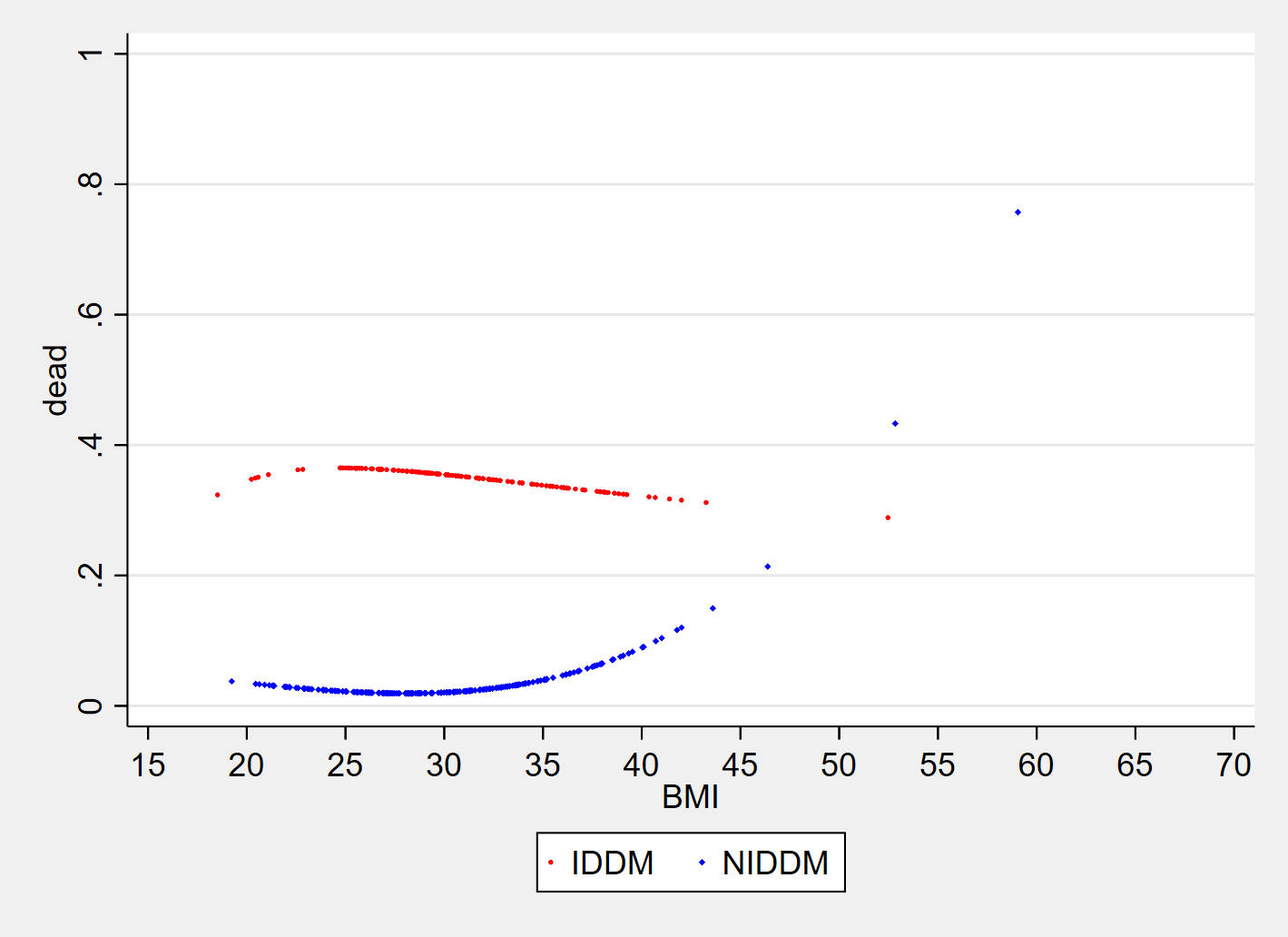


Legend: BMI, body mass index; DM, type 2 diabetes mellitus; HFrEF, heart failure with reduced ejection fraction; IDDM, insulin dependent diabetes mellitus; NIDDM, non insulin dependent diabetes mellitus.

**eFig. 4** Relationship between BMI and 5-year all-cause mortality in ambulatory patients with chronic stable HFrEF and concomitant DM (diabetes subgroup) stratified by HbA1c after multivariable adjustment


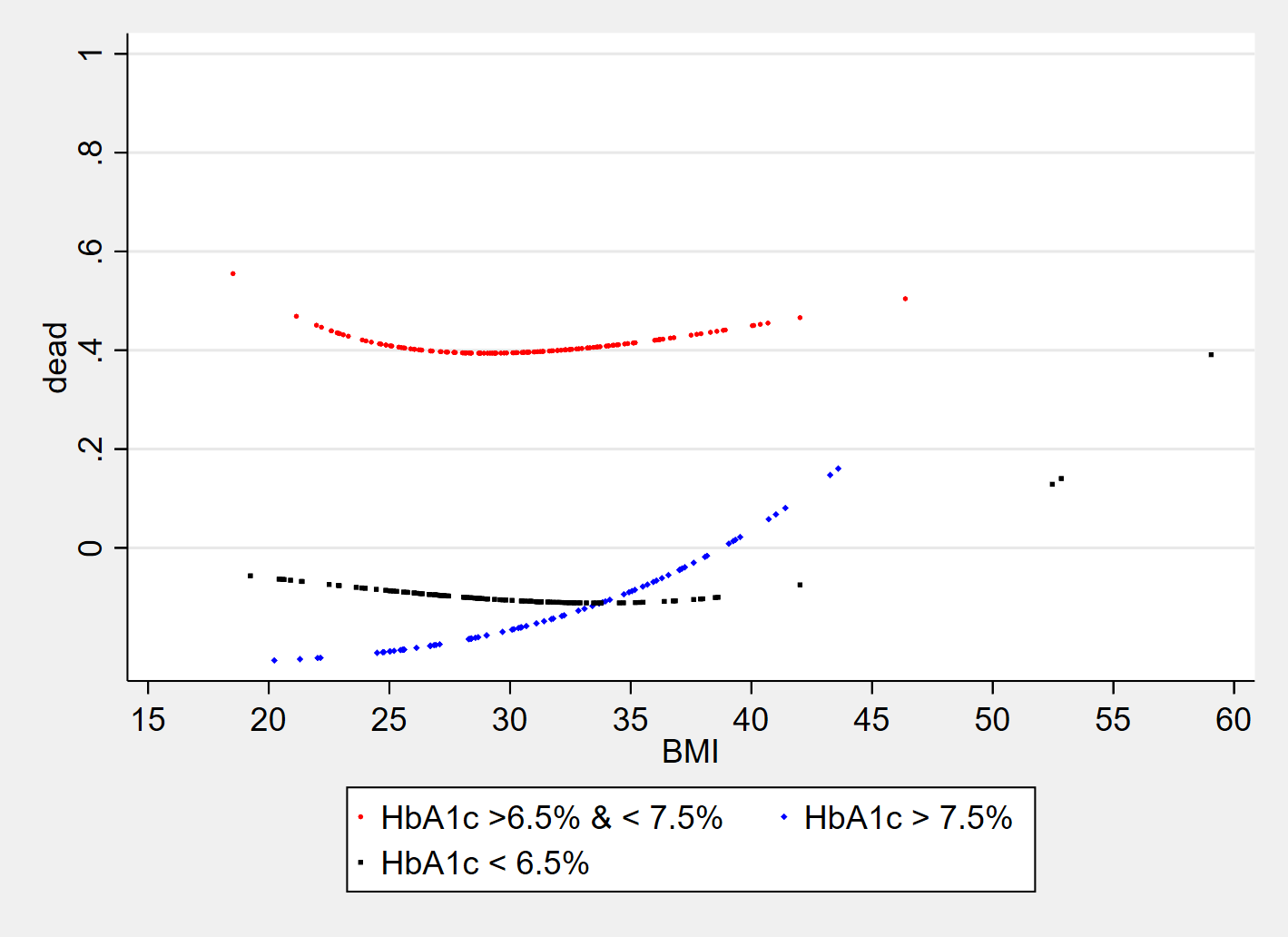


Legend: BMI, body mass index; DM, type 2 diabetes mellitus; HFrEF, heart failure with reduced ejection fraction.

**eFig. 5** Relationship between HbA1c and 5-year all-cause mortality in ambulatory patients with chronic stable HFrEF and concomitant DM (diabetes subgroup) after multivariable adjustment


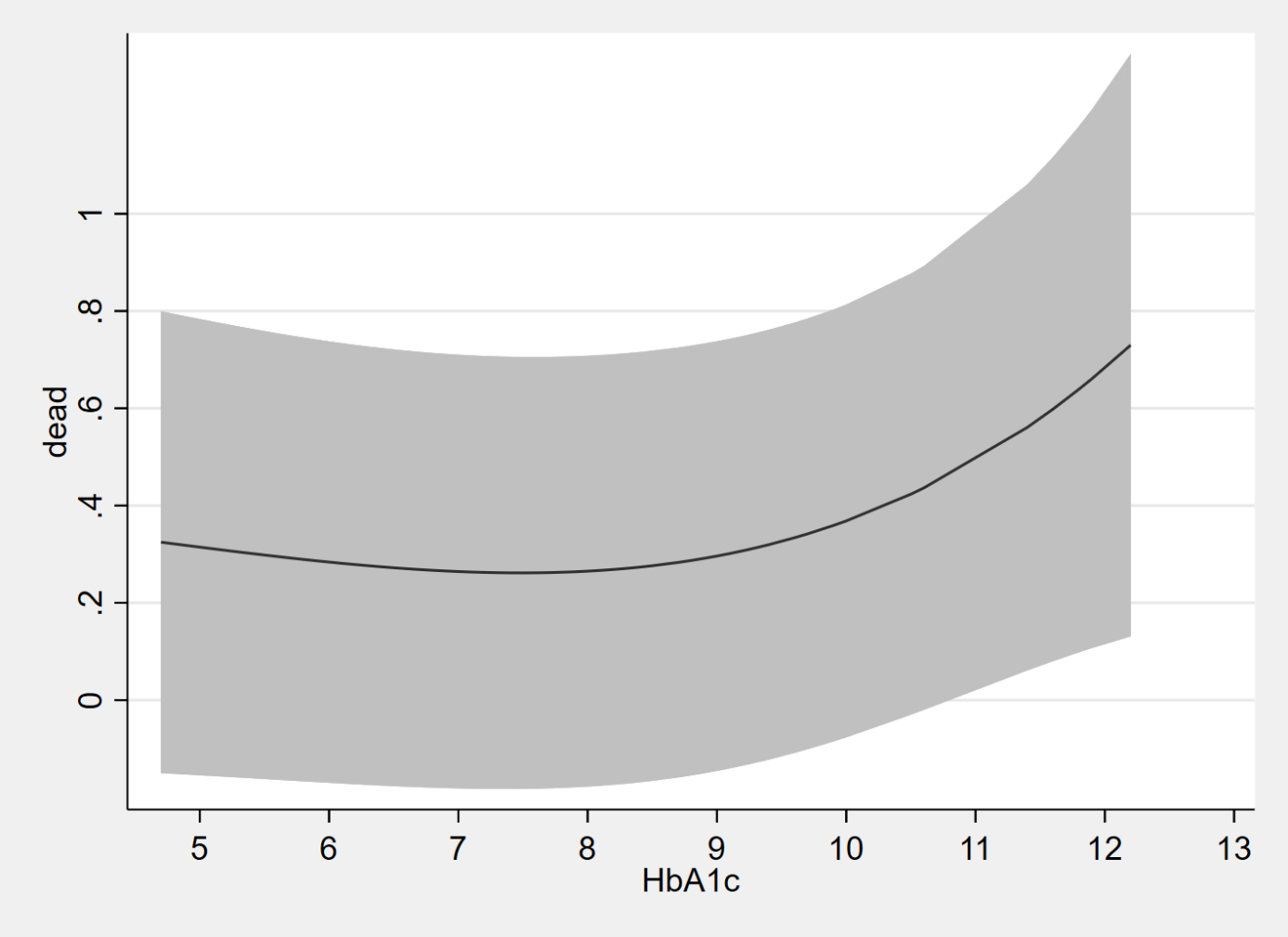


Legend: DM, type 2 diabetes mellitus; HFrEF, heart failure with reduced ejection fraction.

**eFig. 6** Relationship between HbA1c and 5-year all-cause mortality in ambulatory patients with chronic stable HFrEF and concomitant DM (diabetes subgroup) stratified by BMI after multivariable adjustment


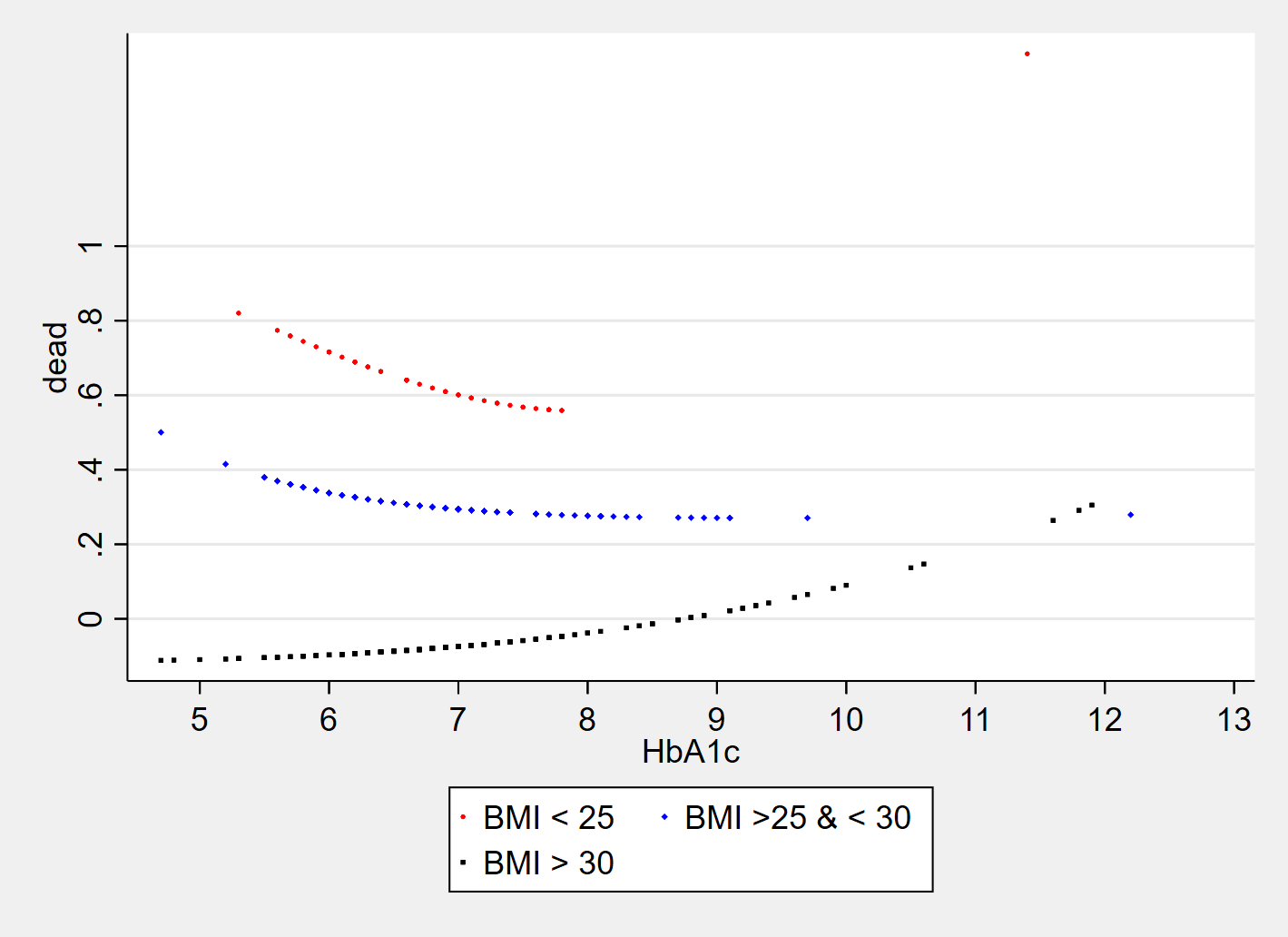


Legend: BMI, body mass index (kg/m²); DM, type 2 diabetes mellitus; HFrEF, heart failure with reduced ejection fraction.
